# Health state utility values (QALY weights) for Huntington’s disease: an analysis of data from the European Huntington’s Disease Network (EHDN)

**DOI:** 10.1007/s10198-019-01092-9

**Published:** 2019-08-13

**Authors:** Annie Hawton, Colin Green, Elizabeth Goodwin, Timothy Harrower

**Affiliations:** 1 Health Economics Group, University of Exeter Medical School, University of Exeter, South Cloisters, St Luke’s Campus, Magdalen Road, Exeter, Devon EX1 2LU UK; 2 Neurology, Royal Devon and Exeter NHS Foundation Trust, Barrack Road, Exeter, Devon EX2 5DW UK

**Keywords:** Huntington’s disease, Health state utility values, Quality-adjusted life-years, Cost-effectiveness analysis, I19

## Abstract

**Background:**

Huntington’s Disease (HD) is a hereditary neurodegenerative disorder which affects individuals’ ability to walk, talk, think, and reason. Onset is usually in the forties, there are no therapies currently available that alter disease course, and life expectancy is 10–20 years from diagnosis. The gene causing HD is fully penetrant, with a 50% probability of passing the disease to offspring. Although the impacts of HD are substantial, there has been little report of the quality of life of people with the condition in a manner that can be used in economic evaluations of treatments for HD. Health state utility values (HSUVs), used to calculate quality-adjusted life-years (QALYs), are the metric commonly used to inform such healthcare policy decision-making.

**Objectives:**

The aim was to report HSUVs for HD, with specific objectives to use European data to: (i) describe HSUVs by demographic and clinical characteristics; (ii) compare HSUVs of people with HD in the UK with population norms; (iii) identify the relative strength of demographic and clinical characteristics in predicting HSUVs.

**Methods:**

European Huntington’s Disease Network REGISTRY study data were used for analysis. This is a multi-centre, multi-national, observational, longitudinal study, which collects six-monthly demographic, clinical, and patient-reported outcome measures, including the SF-36. SF-36 scores were converted to SF-6D HSUVs and described by demographic and clinical characteristics. HSUVs from people with HD in the UK were compared with population norms. Regression analysis was used to estimate the relative strength of age, gender, time since diagnosis, and disease severity (according to the Total Function Capacity (TFC) score, and the UHDRS’s Motor score, Behavioural score, and Cognition score) in predicting HSUVs.

**Results:**

11,328 questionnaires were completed by 5560 respondents with HD in 12 European countries. Women generally had lower HSUVs than men, and HSUVs were consistently lower than population norms for those with HD in the UK, and dropped with increasing disease severity. The regression model significantly accounted for the variance in SF-6D scores (*n* = 1939; *F* [7,1931] = 120.05; *p* < 0.001; adjusted R-squared 0.3007), with TFC score, Behavioural score, and male gender significant predictors of SF-6D values (*p* < 0.001).

**Conclusion:**

To our knowledge, this is the first report of HSUVs for HD for countries other than the UK, and the first report of SF-6D HSUVs described for 12 European countries, according to demographic and clinical factors. Our analyses provide new insights into the relationships between HD disease characteristics and assessment of health-related quality of life in a form that can be used in policy-relevant economic evaluations.

**Electronic supplementary material:**

The online version of this article (10.1007/s10198-019-01092-9) contains supplementary material, which is available to authorized users.

## Background

Huntington’s Disease (HD) is a hereditary neurodegenerative disorder which results in a number of motor, cognitive, and psychiatric symptoms. The condition is characterised by involuntary movements, known as chorea, and an impairment of voluntary movements. Learning and memory problems, depression, obsessive compulsive disorder, and psychosis are frequently reported, as are personality and behaviour changes including apathy, irritability, impulsivity, and obsessionality [1].

HD onset usually occurs when individuals are in their forties, there are no therapies currently available that alter the disease course, and people with HD tend to live only 10–20 years from diagnosis. The gene which causes HD is fully penetrant [2], and it has a devastating effect on an individual’s ability to walk, think, talk and reason [1]. In addition, individuals with HD have a 50% chance of passing the disease onto their children [3]. Diagnosis can have major implications which affect not only the person with HD, but also the rest of the family who may also be at risk of developing the disease [4].

The disorder is relatively uncommon. For example, in 2012, a systematic review and meta-analysis estimated that there was an overall prevalence of adults with HD in Europe, North America, and Australia of 5.7 per 100,000 people [5]. Hence, despite its devastating impact on affected families, HD receives little attention at a national, or international, level. For example, the National Institute for Health and Care Excellence (NICE) [6], which provides guidelines for resource allocation regarding health and social care in the UK, makes no specific reference to the funding of treatments for people with HD.

The breadth of the personal, social, and economic impact of HD has, to date, been under-explored. Research by Busse et al. [7], utilising data from the European Huntington’s Disease Network (EHDN), has been fundamental to starting to establish the health, social, and economic impacts of HD. This work has estimated the proportions of people with HD who use formal and informal care services, and has explored relationships between resource use, disease severity and functional ability. However, to date, no research is apparent which has reported the health-related quality of life (HRQoL) of people with HD in a manner that can be used in policy-relevant economic evaluations of treatments for the condition.

Health outcomes, in the context of health policy decision-making, are frequently considered in the form of quality-adjusted life-years (QALYs). QALYs combine quantity and quality of life in a single outcome measure, whereby length of life is adjusted by a weight representing the quality of life during that time. These quality weights, which are often referred to as health state utility values (HSUVs), can be estimated from preference-based measures [8]. Two of the most commonly used preference-based measures internationally are the EQ-5D [9] and the SF-6D [10].

We conducted a systematic review of HSUVs for HD. This identified just two relevant studies [11] [12]. Research by Hocaoglu et al. [11] focussed on exploring patient-proxy ratings of HRQoL at different stages of HD and investigated the factors which affect proxy ratings, whilst the study by Calvert et al. [12] considered the impact of rare long-term neurological conditions, including HD, on people’s HRQoL. Both studies were conducted in the UK and included samples of 105 and 53 people with HD, respectively. The EQ-5D was used in both studies and no breakdown was provided by demographic or clinical characteristics or disease staging. As such, very little is documented about HSUVs of people with HD.

## Aims

The aims of this research were:

- to describe HSUVs for HD by demographic and clinical characteristics, using data from a European longitudinal, observational study;
- to compare HSUVs of people in the UK with HD with population norms;
- to identify the relative strength of demographic and clinical characteristics in predicting HSUVs for HD.

## Methods

### Participants

Data from the European Huntington’s Disease Network REGISTRY study [13] were used for analysis. This is a multi-centre, multi-national, observational, longitudinal study of people with HD across Europe. Participants complete a six-monthly battery of demographic, clinical, and patient-reported outcome measures, including gender, age, time since diagnosis, disease severity stage according to the Shoulson–Fahn Functional Capacity Rating Scale [14] and motor, cognitive, functional, and behavioural symptoms as rated by the United Huntington’s Disease Rating Scale (UHDRS) [15], and the SF-36. Participants enrolled in the REGISTRY study provide informed, written consent for their anonymised data to be used for research purposes. All those who had provided initial data as of October 2014 were included in the following analyses.

### Measures

#### United Huntington’s Disease Rating Scale (UHDRS) [15]

The UHDRS was developed as a clinical rating scale to assess four domains relating to the clinical features of HD and the course of the disease: motor function, cognitive function, behavioural difficulties, and functional capacity. The measure has undergone extensive reliability and validity testing and has been used as a primary outcome measure in a number of controlled clinical trials. The domains are scored separately, as follows.

##### Total Functional Capacity (TFC)

This rates a person’s independence, using Likert scales, in terms of five domains (occupation, ability to manage finances, ability to perform domestic chores, ability to perform personal activities of daily living, and setting for level of care). The score is the sum of Likert responses (0–3, or 0–2) to five items. Scores range from 0 to 13, with higher scores indicating higher functional capacity. A score of 13/13 is possible on the TFC. This corresponds to functional independence of an individual who has been diagnosed with HD, but has no symptoms.

##### Behavioural score

This is the sum of Likert responses (scored 0–4) which rate the frequency and the severity of 11 behavioural symptoms (e.g., sad/mood, low self-esteem/guilt, disruptive or aggressive behaviour, obsessions, hallucinations, and irritable behaviour). Scores can range from 0 to 88, with higher scores indicative of increased problems.

##### Cognition score

This is the sum of raw scores (number of correct responses) to five cognitive tests. These are a phonemic verbal fluency test (the Controlled Oral Word Association test [16], the Symbol Digit Modalities test [17], and three scores generated from the Stroop Color and Word tests [18]. Higher scores are indicative of better functioning.

##### Motor score

This is the sum of Likert responses (scored 0–4) to 31 motor symptoms items, e.g., ocular pursuit, maximal chorea, gait, and tongue protrusion). Scores range from 0 to 124, with higher scores indicating greater impairment.

#### Shoulson–Fahn Functional Capacity Rating Scale [19]

The TFC score can be used to categorise people into five disease stages defined by the Shoulson–Fahn Functional Capacity Rating Scale. Stage I (earliest stage) includes scores 11–13, Stage II scores 7–10, Stage III scores 3–6, Stage IV scores 1–2, and Stage V score 0 (most advanced stage). Individuals in Stage I may be pre-manifest, i.e., they may have been diagnosed with the condition via genetic testing, but have no clinical expression, Stage II can be characterised by issues with work and some impairment with usual activities, Stage III may include problems with activities of daily living and the requirement for some care support, Stage IV indicates the need for assistance with most activities of daily living and 24 h care may be appropriate, and Stage V is indicative of requiring support with all activities of daily living and progression to the terminal phase of the condition.

#### Sf-36/Sf-6d [20]

The SF-36 includes 36 self-report questions which assess functional health and well-being from the patient’s perspective over the previous 4 weeks [20]. The measure is one of the most widely used tools worldwide for measuring patient-reported outcomes. Participant responses to the SF-36 were converted to SF-6D values using the algorithm provided by Brazier et al. (2002). The SF-6D [10] is a preference-based measure of health, which provides health state utility values. Scores on the SF-6D can range from 0.3 to 1.0, where 0.3 indicates the worst health state and 1.0 the best health state.

### Data analyses

Mean (SD) SF-6D HSUVs were described by gender, age (under 35 years, 35–44 years, 45–54 years, 55–64 years, and 65 years and over), time since diagnosis (1–4 years, 5–9 years, 10 years or more), and disease stage according to the Shoulson–Fahn Functional Capacity Rating Scale (Stage I, Stage II, Stage III, and Stage IV/V). The values were described individually for the 12 European countries and across the countries as a whole. The SF-6D values from people with HD in the UK were compared with SF-6D norms for a representative sample of the UK population, reported by age [21].

A random-effects linear regression analysis was conducted to estimate the relative strength of age, gender, time since diagnosis, and disease severity (according to the Total Functional Capacity score, and the UHDRS’s Motor score, Behavioural score, and Cognition score), in predicting SF-6D values when respondents first provided data to the REGISTRY study. Standardized beta coefficients were computed to directly compare the strength of prediction of each of the independent variables [beta coefficients are regression coefficients obtained by first standardising all variables to a mean of 0 and a standard deviation of 1]. A second regression analysis was conducted as above with the addition of country of residence as 12 binary independent variables, i.e., resident of country X (0) or not resident of country X (1).

Multi-collinearity was assessed by checking correlations between the independent variables, by use of the _rmdcoll in STATA, prior to inclusion in the regression analysis. The adequacy of the model fit was reviewed by examining a normality plot of residuals and a scatterplot of predicted values versus residual values. All analyses were conducted in Excel and STATA 12, and *p* values of less than 0.05 were considered to be statistically significant.

## Results

### Demographic and clinical characteristics

11,328 questionnaires were available from 5560 respondents with HD. Data were provided from more than 12 European countries, including 1510 responses from the UK. The mean (SD) age of respondents was 48.6 (13.3) years and 53% of the sample were female. The mean (SD) time since diagnosis was 4.5 (3.8) years.

Forty-six percent of the respondents were categorised as Stage I in terms of disease progression, 26% Stage II, 21% Stage III, and 7% Stage IV/V. The mean (SD) Total Functional Capacity (TFC) score was 9.04 (3.82), the mean (SD) Behavioural score 12.76 (11.78), the mean (SD) Cognition score 187.49 (85.69), and mean (SD) motor score 29.06 (23.33).

### SF-6D health state utility values according to demographic and clinical characteristics

**Fig. 1:**
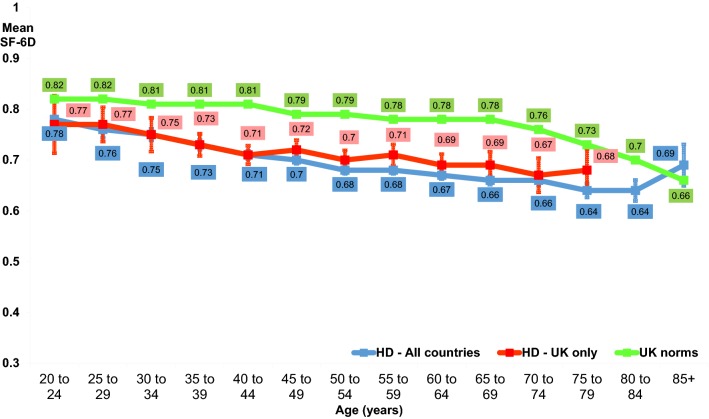
Mean SF-6D health state utility values by age group

**Fig. 2:**
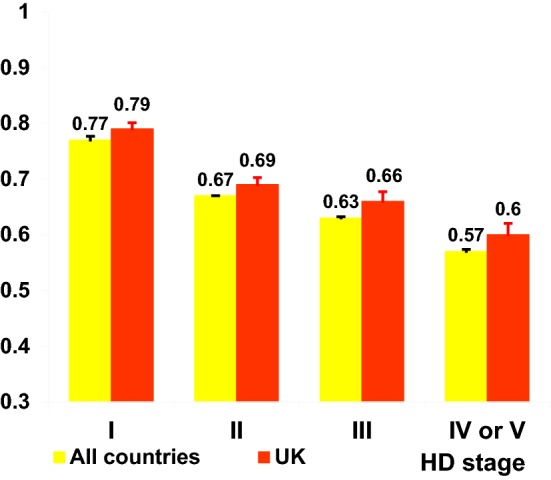
Mean SF-6D health state utility values by disease stage

Ten thousand nine hundred and twenty-seven unique SF-6D scores were available from respondents from all the countries that the REGISTRY includes, with 10,897 SF-6D HSUVs specific to 12 countries. Unadjusted HSUVs are described below and presented in Tables 1, 2, 3, and 4, Figs. 1 and 2.

**Table 1:** SF-6D health state utility values by gender

	Mean	Standard deviation	Min	Max	Observations	Individuals
All countries						
Female	0.695054	0.144963	0.3	1	5774	2836
Male	0.707324	0.140701	0.3	1	5153	2549
Austria						
Female	0.752955	0.137642	0.35	1	88	51
Male	0.783546	0.136137	0.42	1	110	58
France						
Female	0.647912	0.135184	0.3	1	795	360
Male	0.666512	0.138597	0.3	1	691	324
Germany						
Female	0.695595	0.149368	0.3	1	1135	571
Male	0.701092	0.134925	0.32	1	1026	497
Italy						
Female	0.675282	0.129783	0.3	1	248	161
Male	0.700493	0.12746	0.32	1	223	146
Netherlands						
Female	0.71235	0.141317	0.3	1	532	245
Male	0.704964	0.125869	0.41	1	415	182
Norway						
Female	0.726213	0.141851	0.36	1	272	73
Male	0.767959	0.139001	0.39	1	245	85
Poland						
Female	0.696908	0.133193	0.32	1	705	363
Male	0.686235	0.134987	0.3	1	595	315
Portugal						
Female	0.689557	0.156728	0.34	1	384	136
Male	0.708045	0.156622	0.3	1	266	108
Spain						
Female	0.708491	0.150103	0.3	1	636	375
Male	0.722517	0.153181	0.32	1	588	337
Sweden						
Female	0.745081	0.164067	0.33	1	124	52
Male	0.725755	0.144714	0.33	1	139	54
Switzerland						
Female	0.722208	0.13359	0.35	1	77	35
Male	0.699677	0.12578	0.43	0.96	93	37
UK						
Female	0.698362	0.142428	0.3	1	769	405
Male	0.726599	0.136859	0.3	1	741	390

**Table 2:** SF-6D health state utility values by age group

	Mean	Standard deviation	Min.	Max.	Observations	Individuals
All countries						
Under 35 years	0.760387	0.139523	0.3	1	1811	1055
35–44 years	0.717771	0.145022	0.3	1	2660	1451
45–54 years	0.687817	0.138592	0.3	1	2822	1557
55–64 years	0.674979	0.135299	0.3	1	2326	1211
65 years and over	0.658051	0.136079	0.3	1	1308	700
Austria						
Under 35 years	0.811	0.102387	0.64	1	20	13
35–44 years	0.809275	0.156283	0.35	1	69	34
45–54 years	0.736078	0.136427	0.42	1	51	36
55–64 years	0.756061	0.102681	0.53	1	33	17
65 years and over	0.716	0.113284	0.51	0.93	25	16
France						
Under 35 years	0.730351	0.148846	0.36	1	171	86
35–44 years	0.670348	0.13492	0.3	1	345	175
45–54 years	0.641503	0.125873	0.3	1	386	205
55–64 years	0.648115	0.133238	0.3	1	382	192
65 years and over	0.615297	0.132804	0.34	1	202	113
Germany						
Under 35 years	0.774379	0.131078	0.38	1	338	203
35–44 years	0.721186	0.148078	0.33	1	531	294
45–54 years	0.692475	0.135764	0.3	1	602	339
55–64 years	0.659823	0.126737	0.3	1	451	239
65 years and over	0.626276	0.131203	0.32	1	239	112
Italy						
Under 35 years	0.695273	0.137354	0.38	1	55	46
35–44 years	0.699573	0.131722	0.37	0.97	117	81
45–54 years	0.669833	0.131667	0.32	1	120	84
55–64 years	0.681226	0.110028	0.35	0.93	106	72
65 years and over	0.69863	0.139695	0.3	1	73	43
Netherlands						
Under 35 years	0.739927	0.127738	0.39	1	137	75
35–44 years	0.731773	0.148347	0.41	1	203	104
45–54 years	0.704164	0.126814	0.32	1	269	142
55–64 years	0.700437	0.128704	0.41	1	229	108
65 years and over	0.658624	0.132249	0.3	0.97	109	59
Norway						
Under 35 years	0.766897	0.115017	0.55	1	58	26
35–44 years	0.775417	0.142899	0.38	1	120	50
45–54 years	0.731018	0.156396	0.36	1	167	59
55–64 years	0.724952	0.146842	0.39	1	103	43
65 years and over	0.744928	0.104031	0.51	1	69	23
Poland						
Under 35 years	0.740701	0.145356	0.3	1	385	217
35–44 years	0.700069	0.128252	0.34	1	289	165
45–54 years	0.66278	0.124237	0.32	0.96	259	147
55–64 years	0.658906	0.120341	0.4	1	256	138
65 years and over	0.646847	0.102877	0.38	0.96	111	67
Portugal						
Under 35 years	0.789608	0.143568	0.42	1	153	59
35–44 years	0.691326	0.153885	0.37	1	181	73
45–54 years	0.662026	0.147543	0.36	1	153	69
55–64 years	0.645833	0.133548	0.34	1	96	48
65 years and over	0.655224	0.160022	0.3	0.96	67	32
Spain						
Under 35 years	0.794508	0.130217	0.33	1	244	177
35–44 years	0.746099	0.144008	0.3	1	323	208
45–54 years	0.687599	0.1457	0.34	1	279	180
55–64 years	0.667126	0.153473	0.3	1	247	129
65 years and over	0.640992	0.132989	0.38	1	131	86
Sweden						
Under 35 years	0.763571	0.142834	0.34	0.89	28	16
35–44 years	0.762245	0.134788	0.54	1	49	22
45–54 years	0.743253	0.154723	0.33	1	83	35
55–64 years	0.719385	0.17946	0.33	1	65	27
65 years and over	0.686579	0.128237	0.43	1	38	20
Switzerland						
Under 35 years	0.785517	0.119779	0.54	1	29	14
35–44 years	0.691852	0.142452	0.45	0.93	27	17
45–54 years	0.673778	0.116097	0.43	0.92	45	25
55–64 years	0.681225	0.117874	0.35	0.93	49	19
65 years and over	0.776	0.122018	0.54	0.93	20	8
UK						
Under 35 years	0.756667	0.146746	0.3	1	183	114
35–44 years	0.714822	0.140459	0.35	1	394	219
45–54 years	0.712357	0.138297	0.32	1	403	231
55–64 years	0.702671	0.134274	0.3	1	307	177
65 years and over	0.684036	0.13906	0.3	1	223	120

**Table 3:** SF-6D health state utility values by time since diagnosis

	Mean	Standard deviation	Min.	Max.	Observations	Individuals
All countries						
Under 1 year	0.690792	0.14013	0.32	1	1401	1323
1–4 years	0.683108	0.13482	0.3	1	3758	2255
5–9 years	0.659088	0.13342	0.3	1	2346	1388
10 years or more	0.639765	0.12777	0.3	1	767	449
Austria						
Under 1 year	0.744286	0.14587	0.35	1	28	28
1–4 years	0.786868	0.12872	0.4	1	83	51
5–9 years	0.746444	0.12619	0.49	1	45	30
10 years or more	0.653636	0.11281	0.49	0.8	11	10
France						
Under 1 year	0.665258	0.12956	0.36	1	194	184
1–4 years	0.652803	0.12739	0.3	1	610	355
5–9 years	0.622319	0.13019	0.3	1	401	233
10 years or more	0.571809	0.09988	0.3	0.85	94	55
Germany						
Under 1 year	0.684045	0.14096	0.32	1	267	255
1–4 years	0.676967	0.12866	0.32	1	755	464
5–9 years	0.659446	0.13117	0.3	1	469	297
10 years or more	0.649392	0.14043	0.3	1	181	108
Italy						
Under 1 year	0.683171	0.12795	0.37	1	82	78
1–4 years	0.696809	0.12584	0.32	1	188	134
5–9 years	0.652072	0.12413	0.35	0.97	111	78
10 years or more	0.647407	0.15934	0.3	1	27	18
Netherlands						
Under 1 year	0.701318	0.13212	0.32	1	129	120
1–4 years	0.691862	0.12623	0.3	1	333	197
5–9 years	0.667908	0.11375	0.36	1	196	111
10 years or more	0.65	0.10821	0.41	1	60	36
Norway						
Under 1 year	0.731404	0.14487	0.41	1	57	53
1–4 years	0.740581	0.13607	0.38	1	172	79
5–9 years	0.712447	0.14251	0.38	1	94	40
10 years or more	0.701892	0.10173	0.56	1	37	13
Poland						
Under 1 year	0.68426	0.126	0.38	1	277	263
1–4 years	0.664209	0.12526	0.32	1	449	267
5–9 years	0.649503	0.11622	0.38	0.97	201	113
10 years or more	0.624286	0.09676	0.4	0.93	49	28
Portugal						
Under 1 year	0.660678	0.1595	0.36	1	59	53
1–4 years	0.670094	0.15398	0.3	1	213	114
5–9 years	0.631615	0.14723	0.34	1	130	70
10 years or more	0.618438	0.13716	0.36	1	32	16
Spain						
Under 1 year	0.691752	0.15795	0.33	1	137	130
1–4 years	0.683433	0.14167	0.38	1	300	210
5–9 years	0.657255	0.13866	0.3	1	255	147
10 years or more	0.608317	0.12365	0.32	0.89	101	61
Sweden						
Under 1 year	0.690556	0.14293	0.46	0.93	18	17
1–4 years	0.733111	0.16335	0.34	1	90	48
5–9 years	0.707778	0.16237	0.33	1	81	39
10 years or more	0.695833	0.18923	0.33	1	12	7
Switzerland						
Under 1 year	0.764	0.11673	0.58	0.89	10	10
1–4 years	0.698269	0.1462	0.35	0.96	52	27
5–9 years	0.676216	0.10782	0.45	0.85	37	23
10 years or more	0.650526	0.1025	0.48	0.92	19	9
UK						
Under 1 year	0.725315	0.15276	0.36	1	143	132
1–4 years	0.692854	0.13305	0.35	1	508	305
5–9 years	0.677901	0.13128	0.3	1	324	205
10 years or more	0.675278	0.12062	0.32	0.92	144	88

**Table 4:** SF-6D health state utility values by disease stage

	Mean	Standard deviation	Min	Max	Observations	Individuals
All countries						
Stage I	0.766802	0.131412	0.3	1	4991	2869
Stage II	0.674682	0.128271	0.3	1	2753	1755
Stage III	0.632616	0.120776	0.3	1	2282	1500
Stage IV or V	0.574574	0.117588	0.3	1	774	571
Austria						
Stage I	0.84662	0.106234	0.57	1	71	43
Stage II	0.794889	0.116163	0.52	1	45	32
Stage III	0.683878	0.124697	0.35	0.96	49	35
Stage IV or V	0.676957	0.137459	0.42	1	23	17
France						
Stage I	0.727054	0.12818	0.41	1	577	305
Stage II	0.647201	0.123765	0.36	1	393	246
Stage III	0.59593	0.113446	0.3	1	398	264
Stage IV or V	0.544159	0.113143	0.3	1	113	93
Germany						
Stage I	0.762888	0.132533	0.38	1	987	588
Stage II	0.683287	0.126591	0.3	1	505	328
Stage III	0.625195	0.115707	0.32	0.96	435	290
Stage IV or V	0.556803	0.109825	0.3	0.95	147	116
Italy						
Stage I	0.73257	0.119209	0.49	1	214	158
Stage II	0.684516	0.123902	0.33	1	124	93
Stage III	0.629579	0.108911	0.32	0.97	95	79
Stage IV or V	0.582162	0.134001	0.3	0.85	37	32
Netherlands						
Stage I	0.772891	0.130058	0.44	1	422	231
Stage II	0.674352	0.107185	0.36	1	239	152
Stage III	0.656305	0.124521	0.3	1	203	128
Stage IV or V	0.606986	0.10237	0.36	0.96	73	49
Norway						
Stage I	0.788699	0.135195	0.38	1	269	99
Stage II	0.716343	0.141254	0.36	1	134	66
Stage III	0.695222	0.1221	0.38	0.96	90	43
Stage IV or V	0.626522	0.117922	0.39	0.88	23	17
Poland						
Stage I	0.758336	0.127962	0.4	1	607	367
Stage II	0.651849	0.113278	0.39	0.97	357	231
Stage III	0.625165	0.102667	0.3	0.96	273	171
Stage IV or V	0.56459	0.090306	0.38	0.76	61	40
Portugal						
Stage I	0.754729	0.139255	0.36	1	387	153
Stage II	0.637239	0.136918	0.38	1	134	78
Stage III	0.588081	0.131201	0.3	1	99	61
Stage IV or V	0.48579	0.123257	0.34	0.8	19	15
Spain						
Stage I	0.786346	0.133099	0.3	1	665	442
Stage II	0.658841	0.126674	0.33	1	207	150
Stage III	0.640556	0.119292	0.3	0.97	198	139
Stage IV or V	0.565308	0.117455	0.32	0.96	130	77
Sweden						
Stage I	0.788807	0.124229	0.51	1	109	56
Stage II	0.730538	0.156486	0.43	1	93	40
Stage III	0.667805	0.163623	0.33	1	41	30
Stage IV or V	0.586875	0.128152	0.33	0.77	16	10
Switzerland						
Stage I	0.777024	0.11373	0.45	1	84	43
Stage II	0.661667	0.107504	0.43	0.85	36	21
Stage III	0.648438	0.118624	0.35	0.96	32	21
Stage IV or V	0.601765	0.089109	0.45	0.76	17	15
UK						
Stage I	0.789353	0.123431	0.39	1	541	332
Stage II	0.689479	0.130967	0.35	1	461	296
Stage III	0.655718	0.121061	0.3	1	348	222
Stage IV or V	0.603048	0.120736	0.3	0.96	105	81

#### Gender

HSUVs are presented in Table 1 by gender for the countries as a whole and for each of the 12 countries separately. The general pattern was for women to have lower SF-6D values than men.

#### Age

Table 2 and Fig. 1 present SF-6D values by age group. The data show a decline in values with age. The comparison of SF-6D values by age for people with HD in the UK with values from a representative sample of the UK general population shows SF-6D values of people with HD to be consistently lower than those without the condition in all age groups.

#### Time since diagnosis

Table 3 shows a negligible drop in values, as time since diagnosis increases.

#### Disease stage

Table 4 and Fig. 2 indicate that SF-6D values decrease as HD progresses, with the most substantial drop between Stage I and Stage II, both for all the countries combined and the UK separately.

### Strength of demographic and clinical characteristics in predicting HSUVs

Checks for multi-collinearity indicated that it was appropriate to include all independent variables in the regression model, and examination of normality plots of the regression residuals and scatterplots of predicted SF-6D and residual values also indicated the appropriateness of this analysis (Appendices 1 and 2).

Table 5 presents the results of the regression analysis. The model significantly accounted for the variance in SF-6D scores (*n* = 1939; *F* [7,1931] = 120.05; *p* < 0.001; adjusted R-squared 0.3007). TFC score, Behavioural score, and male gender were significant predictors of SF-6D values (all at *p* < 0.001). The regression results for these variables indicated the following:

**Table 5:** Results of random-effects GLS regression to explore relationships between demographic and clinical variables and SF-6D health state utility values

SF-6D value	Coefficient	Standard error	*p* > *z*	Lower confidence interval	Upper confidence interval	Beta coefficients^a^
TFC score	0.0091764	0.0011486	0.0000000	0.0069239	0.0114290	0.2328026
Behavioural score	− 0.0047258	0.0002288	0.0000000	− 0.0051744	− 0.0042771	− 0.4053759
Cognition score	0.0000891	0.0000596	0.1350000	− 0.0000278	0.0002060	0.0430957
MOT score	− 0.0002756	0.0002094	0.1880000	− 0.0006862	0.0001350	− 0.0384748
Male gender	0.0188754	0.0052631	0.0000000	0.0085535	0.0291973	0.0686814
Age	− 0.0002946	0.0002174	0.1760000	− 0.0007210	0.0001318	− 0.0270106
Years since diagnosis	0.0014575	0.0008332	0.0800000	− 0.0001766	0.0030917	0.0375215
Constant	0.6527599	0.0233794	0.0000000	0.6069084	0.6986114	

*n *= 1939; *F* [7,1931] = 120.05; *p *< 0.001; Adjusted R-squared 0.3007

^a^Beta coefficients were obtained by standardising all variables to a mean of 0 and a standard deviation of 1, and then including them in the regression analysis

(i) The coefficient estimated for the TFC variable was 0.009, indicating that an one point change in the TFC score (range 0–13) corresponded to a 0.009 change in SF-6D score. For example, a decline in independence for a person with HD from a position of functional independence with a TFC score of 13 (Stage I on the TFC scale) to a state of total dependence with a TFC score of 0 (Stage V on the TFC scale) has, on average, a corresponding decrease of 0.119 in their HSUV (i.e., a change of 13 points on the TFC scale equates to a decrease of 0.119 on the SF-6D [13*0.0091764]).
(ii) The coefficient for Behavioural score was 0.005, indicating that an increase in behavioural difficulties by one point corresponds to a drop in SF-6D score of 0.005. This equates, for example, to an average drop in SF-6D value of 0.416 from having no behavioural difficulties (score of 0) to having the maximum score on the Behavioural scale (score of 88) [88*− 0.0047258]. Given that a minimally important difference of 0.041 on the SF-6D has been reported [22], these drops in SF-6D scores in line with increased behavioural difficulties appear to have distinct clinical relevance.
(iii) On average, men have SF-6D values 0.019 points higher than women.

The beta weights for the independent variables (given in Table 5) show the relative strength of each of these as predictors of SF-6D values. By far, the strongest predictor was the frequency and severity of behaviour problems (Behavioural score, *β* = − 0.405), followed by the degree of functional independence (TFC score, *β* = 0.233). The next strongest predictor of higher HSUVs was male gender (*β* = 0.069), with the other predictors having relatively negligible effects. The results of the regression analysis including country of residence are shown in Appendix 3.

## Discussion

To our knowledge, this is the first report of HSUVs for HD for countries other than the UK, and the first report of SF-6D values described individually for 12 European countries, broken down by demographic and clinical factors.

Our descriptive analysis indicated that women with HD generally had lower SF-6D HSUVs than men, and this finding held when gender was included in a regression analysis which controlled for other demographic and clinical variables. Although the descriptive data indicated a decline in HSUVs by age group, age was not statistically significantly associated with SF-6D values when other characteristics were controlled for. HSUVs declined by disease stage, with the most substantial drop between Stages I and II. This may be indicative of the fact that some individuals are diagnosed in the pre-manifest stage because of family history of the condition and early predictive testing, and experience no symptoms in Stage I [14]. This may also explain why there was not a statistically significant relationship between HSUVs and time since diagnosis, as some individuals may be diagnosed with HD and live for decades without any clinical expression, whilst others may only be diagnosed at the point of experiencing significant symptomatology.

The regression analysis indicated a significant association between HSUVs and behavioural symptoms, e.g., sadness/low mood, low self-esteem/guilt, disruptive or aggressive behaviour, obsessions, hallucinations, and irritable behaviour. This relationship was more substantial than the relationship between disease severity (as assessed by functional independence) and HSUVs, suggestive of the key role that such behavioural symptoms may play in HRQoL. Our analyses indicate the importance of these behavioural symptoms in the context of much less significant relationships between cognitive symptoms and HSUVs and motor symptoms and HSUVs, and reflect that although the clinical diagnosis of HD is based on the presence of the movement disorder, behavioural changes appear to be the most debilitating aspect of the condition [14]. People with HD are eight times more likely to commit suicide than other members of the general population [23], with HD fracturing lives of entire families: economically, socially, and emotionally. The impacts of HD appear far-reaching with a reported increased prevalence of criminal behaviour in males who carry the gene, and the suggestion that this criminal behaviour is probably linked to the personality changes exhibited as a result of the condition, or related to the depressive reactions to the illness coupled with secondary alcohol misuse [24].

The size and coverage of the European Huntington’s Disease Network REGISTRY study data set has been a major asset for this research. It has enabled us to analyse HSUVs by key demographic variables and clinical factors pertinent to the condition across 12 European countries. Clearly, our results are constrained by the particular measures used in the REGISTRY study. The finding that behavioural symptoms were so strongly related to HSUVs, and cognitive and motor symptoms were not, may be indicative of the instruments used to assess these areas of functioning. However, each of these measures forms part of the United Huntington’s Disease Rating Scale (UHDRS) [15], the main internationally recognised tool for assessing the symptoms of HD.

The ongoing, longitudinal data collection of the REGISTRY study provides a unique opportunity for future research to consider the responsiveness of the SF-6D for people with HD over time, and in relation to treatments that are currently in development. Further research may also usefully explore the comparative responsiveness of the SF-6D and the EQ-5D to health-related changes in the lives of people with HD. The EQ-5D is the most common internationally used preference-based measure for estimating QALYs, and is recommended for use by the UK’s National Institute of Health and Care Excellence in health and social care policy decision-making [25]. It would be of value to determine whether changes in the HRQoL of people with HD are more/less likely to be detected by each of these economic evaluation measures, particularly given that the SF-6D provides higher HSUVs than the EQ-5D because the values are estimated using the standard gamble technique and respondents tend to be risk averse. This is evidenced here as our study found females with HD resident in the UK to have a mean (SD) SF-6D score of 0.698 (0.142), whilst males had a mean (SD) score of 0.727 (0.137). In comparison, the two previous studies that included HSUVs of people with HD, both conducted in the UK, reported EQ-5D scores of a mean (standard deviation) of 0.56 (0.35) [range − 0.33 to 1] [11] and a mean (95% confidence interval) of 0.30 (0.19, 0.41) [12]. Thus, the SF-6D may demonstrate lower health gains relative to estimates based on the EQ-5D. The development of the international Enroll-HD platform [26] may provide additional opportunities to address some of these issues.

In conclusion, this research has provided new empirical data on the health state utility values associated with Huntington’s disease, in a format that can inform the cost-effectiveness analyses of treatments for people with HD. The provision of this information, based on assessments across a number of European countries, can aid health policy decision makers, clinicians, and people with HD.

## Electronic supplementary material

Below is the link to the electronic supplementary material.

Supplementary material 1 (DOCX 38 kb)
