# Supplementary material for: Health state utility values (QALY weights) for Huntington’s disease: an analysis of data from the European Huntington’s Disease Network (EHDN)
Source: Eur J Health Econ. 2019 Aug 13;20(9):1335–47. doi: 10.1007/s10198-019-01092-9 (PMC6856291; doi:10.1007/s10198-019-01092-9)
Supplement: Supplementary file 1 — Supplementary material 1 (DOCX 38 kb) [file 10198_2019_1092_MOESM1_ESM.docx]

*Appendix 1: Predicted SF-6D values by residuals*

*Appendix 2: Normality plot of regression residuals*

*Appendix 3: Results of random-effects GLS regression to explore relationships between demographic and clinical variables, including country of residence, and SF-6D health state utility values*

| **SF-6D value** | **Coefficient** | **Standard error** | **P>z** | **Lower confidence interval** | **Upper confidence interval** | **Beta**  **coefficients*** |
| --- | --- | --- | --- | --- | --- | --- |
| TFC score | 0.0100308 | 0.0011453 | 0.000 | 0.0077848 | 0.0122769 | 0.25521540 |
| Behaviour score | 0.0047304 | 0.0002299 | 0.000 | -0.0051812 | -0.0042796 | -0.40447010 |
| Cognition score | 0.0000905 | 0.0000610 | 0.138 | -0.0000292 | 0.0002102 | 0.00438808 |
| MOT score | -0.0000527 | 0.0002074 | 0.799 | -0.0004594 | 0.0003539 | -0.00732930 |
| Male gender | 0.0192421 | 0.0050870 | 0.000 | 0.0092656 | 0.0292186 | 0.07028040 |
| Age | -0.0003616 | 0.0002121 | 0.088 | -0.0007775 | 0.0000543 | -0.03334770 |
| Years since diagnosis | 0.0008574 | 0.0008205 | 0.296 | -0.0007518 | 0.0024666 | 0.02199000 |
| Austria | 0.0308464 | 0.0199294 | 0.122 | -0.0082384 | 0.0699312 | 0.03994200 |
| France | -0.0404044 | 0.0158366 | 0.011 | -0.0714626 | -0.0093462 | -0.10253580 |
| Germany | -0.0276401 | 0.0152725 | 0.070 | -0.0575919 | 0.0023118 | -0.08482020 |
| Italy | 0.0093683 | 0.0222812 | 0.674 | -0.0343288 | 0.0530654 | 0.00992240 |
| The Netherlands | -0.0475241 | 0.0171995 | 0.006 | -0.0812551 | -0.0137931 | -0.08854650 |
| Norway | 0.0222912 | 0.0191300 | 0.244 | -0.0152258 | 0.0598082 | 0.03151280 |
| Poland | -0.0276677 | 0.0158372 | 0.081 | -0.0587270 | 0.0033915 | -0.06926040 |
| Portugal | -0.0415922 | 0.0194695 | 0.033 | -0.0797751 | -0.0034093 | -0.05770150 |
| Spain | -0.0047129 | 0.0161203 | 0.770 | -0.0363273 | 0.0269016 | -0.01072540 |
| Sweden | -0.0199996 | 0.0270435 | 0.460 | -0.0730363 | 0.0330371 | -0.01590170 |
| Switzerland | -0.0182758 | 0.0248428 | 0.462 | -0.0669965 | 0.0304449 | -0.01648560 |
| UK | 0.0184006 | 0.0158969 | 0.247 | -0.0127758 | 0.0495770 | 0.04466740 |
| Constant | 0.6604259 | 0.0256754 | 0.000 | 0.6100723 | 0.7107795 |  |

*Beta coefficients were obtained by standardising all variables to a mean of 0 and a standard deviation of 1, and then including them in the regression analysis.*
